# Real‐world treatment patterns, healthcare resource utilization, and cost among adults with pulmonary arterial hypertension in the United States

**DOI:** 10.1002/pul2.12090

**Published:** 2022-06-08

**Authors:** Lia N. Pizzicato, Vijay R. Nadipelli, Samuel Governor, Jianbin Mao, Stephan Lanes, John Butler, Rebecca S. Pepe, Hemant Phatak, Karim El‐Kersh

**Affiliations:** ^1^ HealthCore Inc. Wilmington Delaware USA; ^2^ Acceleron Pharma Inc., a wholly owned subsidiary of Merck Sharp & Dohme Corp. Cambridge Massachusetts USA; ^3^ University of Nebraska Medical Center Omaha Nebraska USA

**Keywords:** healthcare costs, healthcare resource utilization, pulmonary arterial hypertension, treatment patterns

## Abstract

Treatment for pulmonary arterial hypertension (PAH) has evolved over the past decade, including approval of new medications and growing evidence to support earlier use of combination therapy. Despite these changes, few studies have assessed real‐world treatment patterns, healthcare resource utilization (HCRU), and costs among people with PAH using recent data. We conducted a retrospective cohort study using administrative claims from the HealthCore Integrated Research Database®. Adult members with claims for a PAH diagnosis, right heart catheterization, and who initiated PAH treatment (index date) between October 1, 2015 and November 30, 2020 were identified. Members had to be continuously enrolled in the health plan for 6 months before the index date (baseline) and ≥30 days after. Treatment patterns, HCRU, and costs were described. A total of 843 members with PAH (mean age 62.3 years, 64.2% female) were included. Only 21.0% of members received combination therapy as their first‐line treatment, while most members (54.6%) received combination therapy as second‐line treatment. All‐cause HCRU remained high after treatment initiation with 58.0% of members having ≥1 hospitalization and 41.3% with ≥1 emergency room visit. Total all‐cause costs declined from $15,117 per patient per month at baseline to $14,201 after treatment initiation, with decreased medical costs ($14,208 vs. $6,349) more than offsetting increased pharmacy costs ($909 vs. $7,852). In summary, despite growing evidence supporting combination therapy, most members with PAH initiated treatment with monotherapy. Total costs decreased following treatment, driven by a reduction in medical costs even with increases in pharmacy costs.

## INTRODUCTION

Pulmonary arterial hypertension (PAH) is characterized by pulmonary vascular remodeling resulting in a progressive increase in pulmonary vascular resistance, right heart failure, and premature mortality. The 6th World Symposium on Pulmonary Hypertension (PH) has defined five types of PH: PAH (Group 1); PH due to left heart disease (Group 2); PH due to chronic lung disease and/or hypoxia (Group 3); PH due to pulmonary artery obstruction including chronic thromboembolic PH (Group 4); and PH due to unclear or multifactorial mechanisms (Group 5).^1^ PAH (PH Group 1) is characterized by mean pulmonary artery pressure greater than 20 mmHg, pulmonary arterial wedge pressure less than or equal to 15 mmHg, and pulmonary vascular resistance greater than or equal to three Wood Units measured by right heart catheterization (RHC) at rest and negative evaluation for other precapillary PH Groups 3–5.^1^ Estimates of PAH incidence range between 2.0 and 7.6 cases per million adults per year.^2^, ^3^, ^4^ In general, people with PAH have high comorbidity burden, high rates of premature mortality (approximately 21% after 3 years), low quality of life, and high healthcare resource utilization (HCRU) and costs.^2^, ^5^, ^6^, ^7^, ^8^, ^9^

Treatment of PAH includes medications that target three signaling pathways involved in disease pathogenesis: the nitric oxide, endothelin, and prostacyclin pathways. Phosphodiesterase 5 inhibitors (PDE5is), which include sildenafil and tadalafil, and soluble guanylate cyclase stimulators (sGCs), which include riociguat, target the nitric oxide pathway. Medications that target the endothelin pathway include three endothelin receptor antagonists (ERAs): bosentan, ambrisentan, and macitentan. Prostacyclin and IP receptor agonists target the prostacyclin pathway and are available parenterally through a continuous infusion either intravenously (epoprostenol and treprostinil) or subcutaneously (treprostinil), or via inhalation (iloprost and treprostinil) or orally (treprostinil and selexipag).^10^ Various multiparametric risk assessment tools are used to predict survival in people with PAH and to guide treatment decisions with an aim for achieving a low risk status.^11^, ^12^

Because three signaling pathways can be targeted by the currently available medications, increasing attention has been placed on using combination therapy, the use of two or more classes of drugs simultaneously, for the treatment of PAH.^13^ Before 2015, treatment guidelines for PAH recommended initial monotherapy followed by sequential combination therapy with clinical worsening.^2^, ^14^ In the AMBITION clinical trial, initial combination therapy with an ERA (ambrisentan) plus a PDE5i (tadalafil) in a treatment‐naïve population lowered the risk of clinical failure events (defined as the first occurrence of a composite of death, hospitalization for worsening PAH, disease progression, or unsatisfactory long‐term clinical response), which led the Food and Drug Administration (FDA) to approve the treatment combination in treatment‐naïve people.^15^ Other clinical trials have also noted reductions in clinical worsening and long‐term morbidity among people on combination therapy.^16^, ^17^, ^18^ In 2015, the European Society of Cardiology (ESC)/European Respiratory Society (ERS) issued updated treatment guidelines that noted the benefits of initial combination therapy for low‐ and intermediate‐risk persons which was further highlighted in the 6th World Symposium on PH.^19^, ^20^ Finally, given the complex routes of administration and the potential for severe side effects of parenteral prostacyclins, they are usually reserved for higher risk people with PAH.^21^ The FDA approval of oral treprostinil in 2013 and selexipag in 2015 has allowed for earlier use of prostacyclins in combination therapy.^7^, ^22^, ^23^

Few studies have examined treatment patterns among persons with PAH since release of the 2015 guidelines. To our knowledge, only one study has examined treatment patterns across all available PAH medications after the 2015 guidelines, which was limited to data through March 2017.^23^ That study examined medication adherence and time to discontinuation of the index treatment regimen for newly diagnosed people.^23^ A second study examined medication adherence, healthcare utilization, and cost using data through September 2017, however, the study was limited to people treated with oral prostacyclins.^24^ The purpose of the current study is to examine treatment patterns, HCRU, and cost among health plan members with PAH beginning treatment since the 2015 update of the PAH treatment guidelines.

## METHODS

### Study design and data source

This retrospective study used the HealthCore Integrated Research Database (HIRD®), which includes medical and pharmacy administrative healthcare claims data from 14 geographically diverse commercial health plans with members across the United States.^25^ Member enrollment data, inpatient and outpatient medical care, and outpatient prescription drug use are tracked longitudinally for each member. Researchers accessed data in the format of a limited data set for which data use agreements were in place with the covered entities in compliance with the Health Insurance Portability and Accountability Privacy Rule. Because this study was a secondary data analysis using a limited data set, Institutional Review Board approval was not required in accordance with HealthCore's Federal Wide Assurance.

### Study population

Health plan members with commercial insurance or Medicare Advantage/Supplemental Part D insurance with ≥1 claim for a PAH medication between October 1, 2015 and November 30, 2020 (member identification period) were selected for inclusion in the study. PAH medications included an ERA (ambrisentan, bosentan, or macitentan), PDE5i (sildenafil or tadalafil), prostacyclin and IP receptor agonists (epoprostenol, iloprost, selexipag, or treprostinil), or an sGC (riociguat). We sought to identify newly treated members, and the first PAH medication received during the identification period was set as the index date. Members with claims for a PAH medication at any point before the index date (variable time for each patient with a minimum of 6 months) were excluded. Members were required to be greater than 18 years old on the index date. Additionally, members were required to have at least one inpatient or two or more outpatient claims on two distinct dates with a diagnosis of PH or PAH (International Classification of Diseases, Ninth Revision, Clinical Modification [ICD‐9‐CM] codes 416.0, 416.8, 416.9; International Classification of Diseases, Tenth Revision, Clinical Modification [ICD‐10‐CM] codes I27.0, I27.21, I27.2, I27.20, I27.29, I27.89, I27.9) and at least one claim for a RHC during the 6 months before and including the index date. Finally, members were required to have continuous pharmacy and medical benefit enrollment for at least 6 months before (baseline period) and at least 30 days after the index date. The postindex period ended on the earlier of health plan disenrollment or study end date (December 31, 2020, Figure 1).

**Figure 1:**
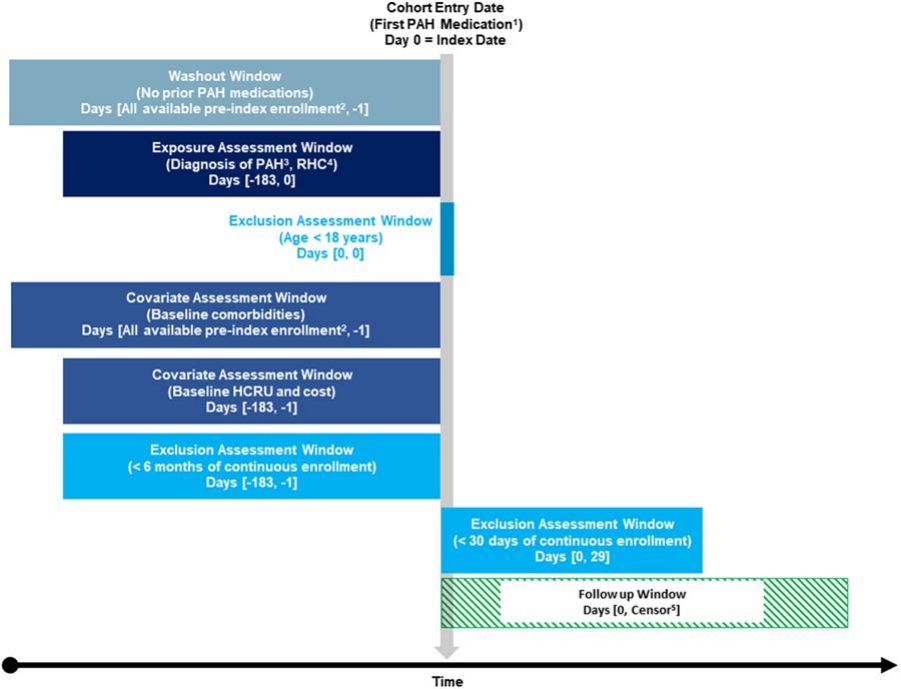
Study design. HCRU, healthcare resource utilization; PAH, pulmonary arterial hypertension; RHC, right heart catherization. ^1^≥1 claim for a PAH medication during the member selection period (October 1, 2015 and November 30, 2020). The first PAH medication is set as the index date. ^2^Defined as member's start of continuous enrollment (variable) in database. ^3^≥1 inpatient or ≥2 outpatient claims on two distinct dates for PAH. ^4^≥1 claim for RHC. ^5^Earliest of disenrollment or end of the study period (December 31, 2020).

### Study measures

Member demographics and clinical characteristics (age, sex, insurance type, prescriber specialty) were assessed on the index date, and the Quan‐Carlson comorbidity index^26^ and other comorbidities were assessed between the health plan enrollment date and index date.

Treatment patterns were assessed during the variable post‐index period. The first line of treatment was determined by the number of different medication classes observed within the first 30 days after the index date, a definition used in prior administrative claims studies.^7^, ^22^, ^23^ Those with only one medication class within the first 30 days were defined as initiating monotherapy, whereas those with two or more medication classes within the first 30 days post‐index were considered to initiate combination therapy. Oral prostacyclin/IP receptor agonists, inhaled prostacyclin, and parenteral prostacyclin were distinguished as three medication classes. The end of the first line of treatment was prompted by either a treatment interruption or modification of treatment. Treatment interruption was defined as a gap in treatment of at least 60 days after the run‐out of days' supply of the last prescription filled. If members had a gap of more than 60 days after the run‐out of days' supply and restarted the same medication after that gap, this would still prompt a new line of treatment. Modification of treatment included the sequential combination of a new medication class or switching medication classes without treatment interruption. The second treatment line started when the member received a new fill for a PAH medication after treatment interruption or at the time of modification of treatment. Treatment patterns were assessed for up to four lines of treatment. Within each line of treatment, medications (by class and drug) and treatment regimens (by class) (e.g., monotherapy ERA, combination ERA + PDE5i) were calculated. A Sankey diagram showing the treatment lines by class of medications for up to four lines of treatment was also created.

All‐cause and PAH‐related HCRU and healthcare costs were assessed during the 6‐month baseline and post‐index periods overall and by each line of treatment. PAH‐related medical visits were defined as claims with an ICD‐9‐CM or ICD‐10‐CM diagnosis of PAH in any position (ICD‐9‐CM codes 416.0, 416.8, 416.9; ICD‐10‐CM codes I27.0, I27.2, I27.20, I27.21, I27.29, I27.89, I27.9), and PAH‐related pharmacy claims were those that included a Healthcare Common Procedure Coding System or a Generic Product Identifier code for a PAH medication. All‐cause and PAH‐related HCRU were calculated as the number and percentage of members with at least one inpatient hospitalization, emergency room visit, outpatient visit, or medication claim (either under the medical or pharmacy benefit). All‐cause and PAH‐related costs were adjusted to 2020 US Dollars (USD) based on the most recent medical price index information provided by the Bureau of Labor Statistics and calculated as the combined health plan paid, member paid, and coordination of benefits costs (third party payer).^27^

### Statistical analysis

Demographic, clinical characteristics, treatment patterns, HCRU, and costs were presented using descriptive statistics. HCRU and costs in US dollars were presented as per patient per month (PPPM) and calculated by taking the sum number of medical and pharmacy claims and costs and dividing it by the total number of PAH patient‐months during the specified time of interest. Frequencies and percentages are provided for categorical variables and means with standard deviations are presented for continuous variables. All analyses were performed using Instant Health Data software (Panalgo).

## RESULTS

### Baseline demographic and clinical characteristics

The study population included 843 health plan members with PAH (Table 1) with a mean age of 62.3 years (standard deviation [SD] = 14.1 years) and 541 (64.2%) were female. Most (59.0%) had commercial insurance as opposed to Medicare Advantage/Supplement/Part D (41.0%), and most were prescribed the first PAH medication by a pulmonologist (43.4%) or cardiologist (25.1%). Members with PAH had a high comorbidity burden with an average Quan‐Charlson comorbidity (QCI) score of 4.6 (SD = 2.7). Common comorbidities included congestive heart failure (75.0%), chronic pulmonary disease (79.8%), and peripheral vascular disease (42.9%). Furthermore, 33.7% were diagnosed with anxiety and 28.2% had depression (Table 2).

**Table 1:** Member selection criteria

Step	Criteria	Member counts	% from previous step
1	Members in the HIRD between October 1, 2015 to November 30, 2020	26,943,277	–
2	From step 1, members with ≥1 claim for a PAH medication^a^ between October 1, 2015 to November 30, 2020; set first PAH medication administration or fill date as the index date	6,431	0.02%
3	From step 2, members ≥18 years old on index date	6,018	94%
4	From step 3, members with ≥1 inpatient or ≥2 outpatient claims on two distinct dates for PAH during 6‐month period before and including the index date	2,452	41%
5	From step 4, members with ≥1 claim for RHC within 6 months before and including the index date	1,127	46%
6	From step 5, exclude members with ≥1 claim for PAH medication before index date (start of continuous enrollment to day [index date ‐ 1])	965	86%
7	From step 6, members with ≥6 months of continuous pharmacy and medical benefit enrollment before index date	870	90%
8	From step 7, members with ≥30 days of continuous pharmacy and medical benefit enrollment after index date	843	97%

*Note*: Member Selection Period: October 1, 2015 to November 30, 2020. Abbreviations: HIRD, HealthCore Integrated Research Database; PAH, pulmonary arterial hypertension; RHC, right heart catherization.

^a^ PAH medications include endothelin receptor antagonists, phosphodiesterase type 5 inhibitors, prostacyclins, and soluble guanylate cyclase stimulators.

**Table 2:** Baseline (6 months) characteristics of PAH study population

	Members
Number of members, *N*	843
Pre‐index duration months, mean (SD)	68.4 (52.7)
Post‐index duration months, mean (SD)	18.5 (15.4)
Age at index (years), mean (SD)	62.3 (14.1)
Female, *n* (%)	541 (64.2%)
Insurance type, *n* (%)	
Commercial	497 (59.0%)
Medicare advantage	200 (23.7%)
Medicare other	146 (17.3%)
Geographic region of member, *n* (%)	
Midwest	274 (32.5%)
Northeast	123 (14.6%)
South	262 (31.1%)
West	184 (21.8%)
Year of index date, *n* (%)	
2015^a^	42 (5.0%)
2016	179 (21.2%)
2017	173 (20.5%)
2018	158 (18.7%)
2019	158 (18.7%)
2020	133 (15.8%)
Specialty of index prescriber, *n* (%)	
Pulmonologist	366 (43.4%)
Cardiologist	212 (25.1%)
PCP	50 (5.9%)
Nonphysician clinician (e.g., PA/NP)	116 (13.8%)
Other/Unknown	99 (11.7%)
QCI, mean (SD)	4.6 (2.7)
Comorbidities	
Congestive heart failure	632 (75.0%)
Peripheral vascular disease	362 (42.9%)
Chronic pulmonary disease	673 (79.8%)
Anxiety	284 (33.7%)
Depression	238 (28.2%)

Abbreviations: N, number; NP, nurse practitioner; PA, physician's assistant; PAH, pulmonary arterial hypertension; PCP, primary care physician; QCI, Quan‐Charlson comorbidity score; SD, standard deviation.

^a^ 2015 includes October 1, 2015 to December 31, 2015 only.

### Treatment patterns

On average, members with PAH were followed for 18.5 months (SD = 15.4) after the index date and had 1.7 (SD = 1.2) lines of treatment (Table 3). In total, 39.7% remained on the first line of treatment throughout the duration of their post‐index period, 29.7% experienced a treatment interruption of 60 days or more while on their first line of treatment, and 30.6% modified their first line of treatment. After the first line, 38.7%, 17.5%, and 9.6% of members began a second, third, and fourth line of treatment, respectively. Among those (*n* = 250) who experienced a treatment interruption of 60 days or more while on the first line, 27.2% eventually restarted treatment with either the same or different medications (Table 3).

**Table 3:** Post‐index treatment patterns among members with PAH by treatment line.^a^

	First line	Second line	Third line	Fourth line
Number of members initiating each line, *N*	843 (100.0%)	326 (38.7%)	148 (17.5%)	81 (9.6%)
Treatment patterns				
Months in treatment line^b^, mean (SD)	7.8 (9.8)	7.4 (9.5)	6.8 (8.1)	6.4 (6.4)
Remain on treatment line through end of postindex^c^, *n* (%)	335 (39.7%)	139 (42.6%)	50 (33.8%)	35 (43.2%)
Treatment interruption^d^, *n* (%)	250 (29.7%)	56 (17.2%)	26 (17.6%)	≤10
Restart treatment after interruption^e^, *n* (%)	68 (27.2%)	17 (30.4%)	≤10	≤10
Do not restart treatment after interruption^e^, *n* (%)	182 (72.8%)	39 (69.6%)	17 (65.4%)	≤10
Members who modify^f^, *n* (%)	258 (30.6%)	131 (40.2%)	72 (48.6%)	36 (44.4%)
Medications in treatment line^g^				
ERA, *n* (%)	238 (28.2%)	163 (50.0%)	80 (54.1%)	51 (63.0%)
Ambrisentan	123 (14.6%)	79 (24.2%)	43 (29.1%)	27 (33.3%)
Bosentan	≤10	0 (0.0%)	0 (0.0%)	0 (0.0%)
Macitentan	117 (13.9%)	85 (26.1%)	38 (25.7%)	24 (29.6%)
PDE5i, *n* (%)	588 (69.8%)	218 (66.9%)	92 (62.2%)	42 (51.9%)
Sildenafil	409 (48.5%)	121 (37.1%)	47 (31.8%)	25 (30.9%)
Tadalafil	201 (23.8%)	106 (32.5%)	47 (31.8%)	17 (21.0%)
Prostacyclin, *n* (%)	118 (14.0%)	100 (30.7%)	57 (38.5%)	44 (54.3%)
Oral	30 (3.6%)	45 (13.8%)	34 (23.0%)	24 (29.6%)
Oral trepostinil	≤10	13 (4.0%)	≤10	≤10
Selexipag	22 (2.6%)	33 (10.1%)	24 (16.2%)	18 (22.2%)
Inhaled	35 (4.2%)	29 (8.9%)	≤10	≤10
Iloprost	0 (0.0%)	≤10	0 (0.0%)	0 (0.0%)
Inhaled treprostinil	35 (4.2%)	28 (8.6%)	≤10	≤10
Parenteral	54 (6.4%)	28 (8.6%)	19 (12.8%)	11 (13.6%)
Treprostinil	26 (3.1%)	17 (5.2%)	14 (9.5%)	≤10
Epoprostenol	28 (3.3%)	11 (3.4%)	≤10	≤10
sGC stimulator (riociguat), *n* (%)	94 (11.2%)	40 (12.3%)	18 (12.2%)	14 (17.3%)
Treatment regimens^h^				
Monotherapy, *n* (%)	664 (78.8%)	148 (45.4%)	67 (45.3%)	30 (37.0%)
ERA	99 (11.7%)	27 (8.3%)	21 (14.2%)	≤10
PDE5i	436 (51.7%)	89 (27.3%)	35 (23.6%)	13 (16.0%)
Oral prostacyclin	≤10	≤10	≤10	≤10
Inhaled prostacyclin	22 (2.6%)	≤10	≤10	≤10
Parenteral prostacyclin	30 (3.6%)	≤10	≤10	≤10
sGC	72 (8.5%)	13 (4.0%)	≤10	≤10
Combination therapy, *n* (%)	179 (21.2%)	178 (54.6%)	81 (54.7%)	51 (63.0%)
ERA + PDE5i	103 (12.2%)	72 (22.1%)	24 (16.2%)	≤10
Other combinations	76 (9.0%)	106 (32.5%)	57 (38.5%)	41 (50.6%)

Abbreviations: ERA, endothelin receptor antagonist; IQR, interquartile range; N, number; PAH, pulmonary arterial hypertension; PDE5i, phosphodiesterase 5 inhibitor; SD, standard deviation; sGC, soluble Guanylate Cyclase stimulator.

^a^ Post‐index period is defined at index date to end of continuous enrollment or study period end (December 31, 2020), whichever comes first.

^b^ Measured as the number of months from treatment initiation to treatment interruption or modification.

^c^ The proportion of members who remain on continuous treatment from index date to the end of post‐index period allowing for a maximum fixed gap of 60 days. Gap refers to the time between the run‐out date of the previous fill, calculated as fill date plus days' supply, and the date of the subsequent fill between index date and day 365.

^d^ The proportion of members who have a gap of >60 days between fills. Gap refers to the time between the run‐out date of the previous fill, calculated as fill date plus days' supply, and the date of the subsequent fill between index date and day 365.

^e^ Among those who have treatment interruption. Member may restart the same treatment or a different treatment after the 60 days.

^f^ Modification occurs when a member switches drug classes (without treatment interruption) or when members adds a sequential combination therapy.

^g^ Medications in treatment line are not mutually exclusive.

^h^ Treatment regimens are mutually exclusive.

While on the first line of treatment, 69.8% of cohort members received a PDE5i, 28.2% an ERA, 14.0% a prostacyclin, and 11.2% an sGC. Use of PDE5is decreased slightly by line of treatment from 69.8% among those who initiated a first line of treatment to 66.9%, 66.2%, and 51.9% among those initiating a second, third, and fourth line of treatment, respectively. While PDE5is declined with each line of treatment, the proportion who used ERAs, prostacyclins, and sGCs tended to increase with each subsequent line of treatment. Specifically, the prevalence of prostacyclin use among those initiating a first, second, third, and fourth line of treatments was 14.0%, 30.7%, 38.5%, and 54.3%, respectively. In general, use of parenteral prostacyclins was low with only 6.4%, 8.6%, 12.8%, and 13.6% using as a first, second, third, and fourth line of treatment, respectively (Table 3).

**Figure 2:**
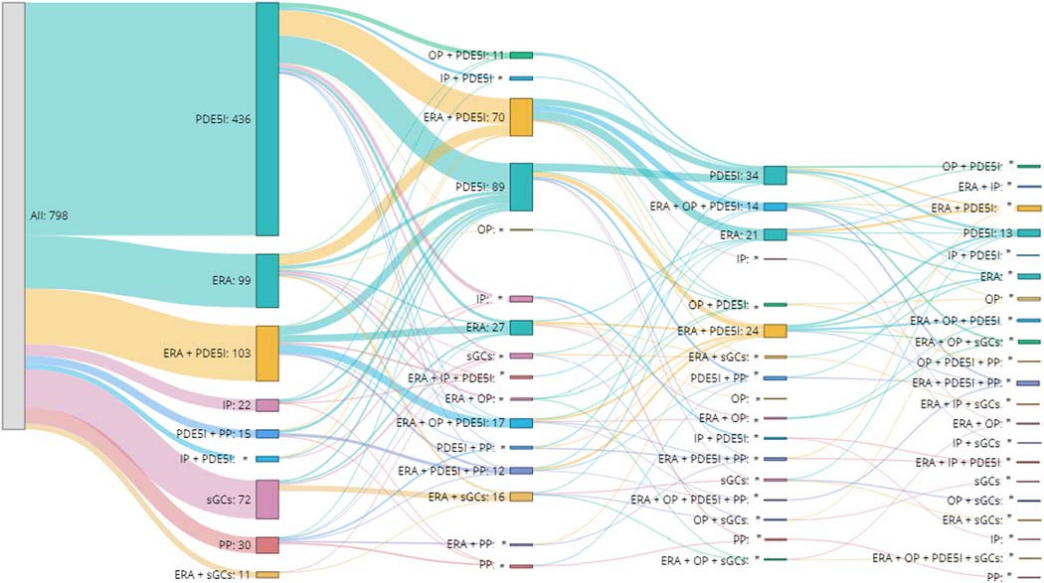
PAH medications by treatment line.^1^ ERA, endothelin receptor antagonist; IP, inhaled prostacyclin; OP, oral prostacyclin; PAH, pulmonary arterial hypertension; PDE5I, phosphodiesterase 5 inhibitor; PP, parenteral prostacyclin; sGCs, soluble guanylate cyclase stimulators.^1^ Sankey diagrams show treatments that comprise ≥1% of total line *n ≤ 10.

For the first line of treatment, most members with PAH were on monotherapy (78.8%) with 51.7% receiving a PDE5i, 11.7% receiving an ERA, and 8.5% receiving an sGC. Only 21.2% of members initiated a combination therapy as the first line of treatment with the most common combination therapy being an ERA + PDE5i, received by 12.2% of members in the cohort. The proportion of the study population on combination therapy increased among those who initiated subsequent lines of treatment to 54.6%, 54.7%, and 63.0% in the second, third, and fourth lines, respectively (Table 3). An ERA + PDE5i combination remained the most prevalent combination through the second line of treatment. The flow of members across lines of treatment is presented in a Sankey diagram (Figure 2).

### Healthcare resource utilization

HCRU was high in both the 6‐month baseline and post‐index period. While the percent of members with at least one all‐cause inpatient hospitalization increased slightly from the baseline to the post‐index period (from 56.0% to 58.0%), the number of inpatient hospitalizations PPPM decreased from 0.17 in the baseline to 0.09 in the post‐index period. All members (100.0%) had an all‐cause outpatient visit in the 6‐month baseline, and nearly all (98.8%) had an all‐cause outpatient visit in the post‐index period. All‐cause outpatient visits decreased slightly from 5.84 PPPM in the baseline period to 5.60 visits PPPM in the post‐index period. In total, 4.5% of members had a lung or lung and heart transplant after treatment initiation (Table 4).

**Table 4:** Baseline (6 months) and post‐index healthcare resource utilization overall and by treatment line

	Baseline^a^	Post‐index^b^	First line	Second line	Third line	Fourth line
Number of members, *N*	843	843	843	326	148	81
Number of months in time period, mean (SD)	6.0 (0.0)	18.5 (15.4)	7.8 (9.8)	7.4 (9.5)	6.8 (8.1)	6.4 (6.4)
≥1 claim, *n* (%)						
All causes						
Inpatient hospitalization	472 (56.0%)	489 (58.0%)	313 (37.1%)	114 (35.0%)	40 (27.0%)	27 (33.3%)
Lung or lung/heart transplant		45 (5.3%)	34 (4.0%)	≤10	≤10	0 (0%)
Emergency room visits	212 (25.1%)	348 (41.3%)	199 (23.6%)	63 (19.3%)	24 (16.2%)	18 (22.2%)
Outpatient visits	843 (100.0%)	833 (98.8%)	825 (97.9%)	317 (97.2%)	147 (99.3%)	76 (93.8%)
Pharmacy fills	827 (98.1%)	834 (98.9%)	833 (98.8%)	324 (99.4%)	148 (100%)	80 (98.8%)
PAH related^c^						
Inpatient hospitalization	422 (50.1%)	419 (49.7%)	255 (30.2%)	102 (31.3%)	37 (25.0%)	26 (32.1%)
Emergency room visits	40 (4.7%)	124 (14.7%)	70 (8.3%)	23 (7.1%)	13 (8.8%)	≤10
Outpatient visits	725 (86%)	774 (91.8%)	723 (85.8%)	285 (87.4%)	121 (81.8%)	66 (81.5%)
PAH medication claims^d^						
Medical administration		109 (12.9%)	72 (8.5%)	42 (12.9%)	25 (16.9%)	13 (16.0%)
Pharmacy fills		819 (97.2%)	806 (95.6%)	305 (93.6%)	135 (91.2%)	74 (91.4%)
PPPM^e^						
All causes						
Inpatient hospitalization	0.17	0.09	0.10	0.09	0.07	0.07
Emergency room visits	0.07	0.05	0.05	0.04	0.03	0.04
Outpatient visits	5.84	5.60	5.90	5.40	5.81	4.96
Pharmacy fills	2.95	3.86	4.08	4.47	4.24	4.16
PAH‐related^d^						
Inpatient hospitalization	0.11	0.06	0.07	0.07	0.06	0.07
Emergency room visits	0.01	0.01	0.01	0.01	0.02	0.02
Outpatient visits	1.24	1.34	1.57	1.58	1.75	1.75
PAH medication claims						
Medical administration		0.10	0.09	0.13	0.49	0.35
Pharmacy fills		0.85	1.08	1.35	1.27	1.38

Abbreviations: GPI, generic product identifier; HCPCS, Healthcare common procedure coding system; ICD‐10‐CM, International Classification of Diseases, Tenth Revision, Clinical Modification; ICD‐9‐CM, International Classification of Diseases, Ninth Revision, Clinical Modification; N, number; PAH, pulmonary arterial hypertension; PPPM, per patient per month; SD, standard deviation.

^a^ Baseline period is defined as the six‐month period before index date.

^b^ Post‐index period is defined as index date to end of continuous enrollment or study period (whichever comes first).

^c^ PAH medical utilization is based on medical claims with an ICD‐9‐CM or ICD‐10‐CM diagnosis code for PAH.

^d^ Includes ERA, PDE5i, prostacyclin, and sGC medications dispensed under medical (identified via HCPCS codes) or pharmacy benefit (identified via GPI codes).

^e^ PPPM is calculated by summing the total number of claims across members in the cohort during the specified time period and dividing by the sum of total months of enrollment across all members in the cohort during the specified time period.

### Cost

Between the 6‐month baseline and post‐index periods, all‐cause medical costs decreased from $14,208 to $6,349 PPPM, and all‐cause pharmacy costs increased from $909 to $7,852. Even with increases in pharmacy costs, total all‐cause costs decreased from $15,117 to $14,201 PPPM driven by the overall decrease in medical costs. All‐cause total costs increased with each line of treatment. Costs were $15,352 PPPM while on the first line of treatment and subsequently increased to $20,021, $20,891, and $25,038 PPPM on the second, third, and fourth line of treatment, respectively (Table 5).

**Table 5:** Baseline (6 months) and post‐index healthcare costs (in 2020 USD) overall and by treatment line

PPPM Healthcare costs^a^	Baseline^b^	Post‐index^c^	First line	Second line	Third line	Fourth line
Number of members, N	843	843	843	326	148	81
Number of months in time period, mean (SD)	6.0 (0.00)	18.5 (15.41)	7.8 (9.78)	7.4 (9.51)	6.8 (8.11)	6.4 (6.43)
All cause						
Total costs	$15,117	$14,201	$15,352	$20,021	$20,891	$25,038
Medical costs	$14,208	$6,349	$7,480	$6,510	$7,028	$8,074
Pharmacy costs	$909	$7,852	$7,871	$13,510	$13,863	$16,964
PAH‐related^d^						
Total costs	$10,868	$10,506	$11,365	$16,810	$18,420	$23,198
Medical costs	$10,868	$3,617	$4,460	$4,171	$5,604	$7,046
Pharmacy costs		$6,889	$6,905	$12,639	$12,816	$16,152

Abbreviations: COB, coordination of benefits; ERA, endothelin receptor antagonist; ICD‐10‐CM, International Classification of Diseases, Tenth Revision, Clinical Modification; ICD‐9‐CM, International Classification of Diseases, Ninth Revision, Clinical Modification; N, number; PAH, pulmonary arterial hypertension; PDE5i, Phosphodiesterase 5 inhibitor; PPPM, per patient per month; SD, standard deviation; sGC, soluble Guanylate Cyclase stimulator; USD, United States Dollar.

^a^ Costs include plan paid, member paid, and COB (third party payer) and were adjusted to 2020 USD; PPPM is calculated by summing the total costs across members in the cohort during the specified time period and dividing by the sum of total months of enrollment across all members in the cohort during the specified time period.

^b^ Baseline period is defined as the 6‐month period before index date.

^c^ Post‐index period is defined as index date to end of continuous enrollment or study period (whichever comes first).

^d^ PAH medical costs is based on medical claims with an ICD‐9‐CM or ICD‐10‐CM diagnosis code for PAH; PAH pharmacy costs are for ERA, PDE5i, prostacyclin, and sGC medications dispensed under pharmacy benefit.

## DISCUSSION

This study examined real‐world treatment patterns, HCRU, and cost following publication of the 2015 ESC/ERS guidelines, which put forth evidence that physicians could treat people with low‐ and intermediate‐risk PAH with either upfront or sequential combination therapy.^19^ In this study, 21.2% of members with PAH initiated treatment on combination therapy. The findings show a modest increase in use of first‐line combination therapy from previous studies using real‐world data in which between 4% and 10% of cohort members initiated treatment with combination therapy.^7^, ^22^, ^23^, ^28^ Importantly, these studies were largely conducted on administrative data before the 2015 guideline update. In the study by Studer et al., 13.0% of their cohort used combination therapy as a first‐line treatment in the period after guideline update (August 2015 to March 2017).^23^

The 2015 guidelines did not recommend upfront combination therapy over sequential combination therapy in people with low‐ to intermediate‐risk PAH and instead provided evidence supporting either treatment strategy.^19^ In our study, if sequential combination occurred more than 30 days after the index date, the first line of treatment would have been classified as monotherapy. A sequential combination therapy treatment strategy may, in part, explain lower than expected utilization of combination therapy as a first line of treatment in the present study. In 2019, the American College of Chest Physicians (CHEST) Guideline and Expert Panel Report on Pharmacotherapy recommended upfront combination therapy over monotherapy for treatment‐naïve people with a WHO functional class (FC) II or III provided the person is willing and able to tolerate it.^29^ The recommendations noted that some people may be unwilling to use combination therapy as it has higher costs and can increase risk for adverse events.^29^ Additionally, the 6th World Symposium on Pulmonary Hypertension published guidelines in 2019 indicating the treatment strategy should be guided by a risk stratification approach.^20^ Specifically, for people with low‐ or intermediate‐risk PAH, combination therapy should be used with only a residual role for monotherapy in specific subsets of people with PAH in whom the efficacy or safety of initial combination therapy was not established.^20^ Because this study included less than 2 years of data since publication of these guidelines, further research is needed to understand the impact of these more recent guidelines on real‐world treatment patterns.

The most common first‐line combination therapy in this study was an ERA + PDE5i with 57.5% of members in the cohort receiving this regimen. According to the 2015 ESC/ERS guidelines and more recent 2019 CHEST guidelines, the combination with the greatest evidence base for treatment of WHO FC II and III is ambrisentan (ERA) and tadalafil (PDE5i).^19^, ^29^ Although this study did not look at specific medications so it is unknown if members had this specific combination of drugs, the high prevalence of the ERA + PDE5i combination may be in part driven by these recommendations. For people on an established PAH therapy, there are several recommendations for sequential combination therapy in the 2015 ESC/ERS guidelines and upheld by the 6th World Symposium on Pulmonary Hypertension, which include macitentan (ERA) added to sildenafil (PDE5i), riociguat (sGC) added to bosentan (ERA), selexipag (oral prostacyclin) added to an ERA or PDE5i, and sildenafil (PDE5i) added to epoprostenol (parenteral prostacyclin).^19^, ^20^ Among members who initiated a second‐line combination therapy (*n* = 178) in the present study, the most prevalent treatment combination remained an ERA + PDE5i (40.5%). The high prevalence of ERA + PDE5i as the most prevalent first‐ and second‐line combination is also consistent with previous studies.^5^, ^7^, ^22^

The prevalence of prostacyclin use among members in this cohort initiating a first or second line of treatment was 14.0% and 30.7%, respectively, which is higher than a previous study in which only 8.1% and 22.4% of the cohort used a prostacyclin as a first and second line of treatment, respectively.^7^ The prior study used data predating the approval of selexipag in 2015, and the higher prevalence of prostacyclin use in our study appears to be driven by selexipag. This result aligns with the 2015 ESC/ERS guidelines, which recommended selexipag as monotherapy or added to an ERA and/or PDE5i as sequential therapy for WHO‐FC II or III.^19^

Riociguat, an sGC, was approved to treat PAH by the FDA in 2013. In our cohort, 11.2% of members used riociguat as a first‐line treatment. This is markedly higher than prior studies in which between 0% and 5% of studied cohorts used an sGC as a first‐line treatment, although these prior studies spanned years that included time before 2013 when riociguat was approved.^7^, ^22^, ^23^ In a systematic review of interventions for PAH, monotherapy riociguat and combination ERA + PDE5i ranked best at reducing clinical worsening followed by monotherapy PDE5i and monotherapy ERA.^30^ Importantly, the estimate for riociguat was based on a single study, so this should be interpreted with caution.^30^

Nearly 30% of members with PAH in the present study had a treatment interruption to their first line of treatment lasting at least 60 days. Although different definitions of treatment interruption have been used (e.g., 30‐ and 90‐day gaps), this result is a slight improvement compared with previous studies in which between 37% and 38% of people had a treatment interruption.^7^, ^22^, ^31^ Factors driving high rates of interruption may include misdiagnosis, intolerable side effects, and high medication costs.^7^

Hospitalization is an important measure of clinical worsening among people with PAH.^32^ The Registry to Evaluate Early and Long‐Term PAH Disease Management (REVEAL) risk calculator (REVEAL 2.0) is used to help physicians make treatment decisions based on an individual's risk profile.^11^ All cause hospitalization within the previous 6 months was added to the calculation such that those with prior hospitalizations will have greater REVEAL 2.0 risk scores.^11^ Future studies may consider examining the role of hospitalization in real‐world treatment intensification and decision making. Although the hospitalization rate decreased after treatment initiation, the proportion of members hospitalized remained high with 58.0% of members experiencing at least one hospitalization and 49.7% of members having a PAH‐related hospitalization. High rates of hospitalization were also found in the REVEAL Registry where 56.8% of people in the registry had at least one hospitalization over the 3‐year post‐index period and in a study by Studer et al. in which 66.4% of the cohort had a hospitalization in the post‐index period.^7^, ^33^ As hospitalization has been associated with increased mortality and is an important determinant of medical costs among people with PAH,^5^, ^31^, ^33^ further understanding of the impact of different treatment patterns on hospitalization outcomes would help support and refine treatment guidelines.

Findings from the present study underscore the high healthcare cost associated with PAH. Total all‐cause and PAH‐related costs were $14,201 PPPM and $10,506 PPPM, respectively, with costs increasing with each subsequent line of treatment. Even with adjustments for inflation, all‐cause costs are higher in the present study compared to prior studies.^5^, ^22^, ^28^, ^31^ For example, Sikirica et al. found all‐cause costs to be $8,187 per month (2011 USD), Copher et al. found all‐cause costs to be $9,295 PPPM (2008 USD), Angalakuditi et al. found PAH all‐cause costs to be $3,236 PPPM (2008 USD), and Burger et al. found all‐cause costs to be $8,987 PPPM (2014 USD).^5^, ^22^, ^28^, ^31^ The higher costs in the present study may be due in part to member selection criteria (e.g., our study required a PAH medication and RHC for inclusion) but may also be related to increased utilization of high cost specialty medications such as riociguat and selexipag. All‐cause medical costs decreased after initiating treatment, a finding mirrored in studies by Burger et al. and Sikirica et al.^22^, ^31^ In addition, the offset resulted in a net decrease in total costs, a finding also seen by Sikirica et al.^31^ This suggests that the high cost of PAH medications may be offset by reductions in medical costs associated with treating PAH such as decreasing hospitalizations, a hypothesis that requires further research to evaluate.

The findings should be interpreted with consideration of several limitations. First, administrative claims are collected for the purpose of payment and not for research and may not reflect true diagnoses and treatment as coding issues may occur and medications may not be taken as prescribed. Some of the ICD‐9‐CM and ICD‐10‐CM diagnosis codes used to identify members with PAH were for PH and not PAH specifically, so it is possible we inadvertently captured members with PH Groups 2–5 in our definition. We increased specificity of identifying members with PAH by also requiring medications for PAH and an RHC as inclusion criteria,^34^, ^35^, ^36^ but riociguat is used to treat both PH group 1 (i.e., PAH) and PH group 4 disease. Second, important member demographics, clinical characteristics, and data on health‐related quality of life are not available in medical and pharmacy claims. For example, functional class, exercise capacity, and echocardiogram results are important measures used in risk assessments and to guide treatment decisions, but these data are unavailable in claims.^19^, ^20^, ^29^ Third, it is possible that the definition of index treatment (i.e., evidence of two or more classes of PAH medications within the first 30 days after index date) was too rigorous and may not account for slower uptake of combination therapy. For example, providers may want to monitor tolerability of a medication before adding a second, or delays in prior authorization may delay uptake of combination therapy. Combination therapy was defined similarly to previous studies that have examined treatment patterns among people with PAH.^7^, ^22^, ^23^ Fourth, follow‐up time for some members occurred during the COVID‐19 pandemic, which may have impacted treatment patterns and outcomes. Finally, study results may not be generalizable to the overall PAH population because people who have commercial and Medicare insurance may have different characteristics than those who are uninsured or on Medicaid.

This study provides a real‐world perspective on treatment patterns, HCRU, and cost following changing treatment guidelines and approval of new medications. Most people initiated treatment on monotherapy during our study period even with growing evidence to support initiating treatment with combination therapy. HCRU remained high following treatment initiation although the number of all‐cause inpatient stays, emergency room visits, and outpatient visits PPPM decreased from the baseline to the postindex period. Initiation of treatment reduced total all‐cause spending even with increases in pharmacy costs. These findings highlight the value of real‐world studies in examining treatment patterns and outcomes among people with PAH. Future studies should compare effectiveness of different treatment modalities (e.g., upfront monotherapy vs. combination therapy) on HCRU and cost.

## AUTHOR CONTRIBUTIONS

Lia N. Pizzicato designed the study and prepared the first draft of the protocol. Vijay R. Nadipelli, Samuel Governor, Stephan Lanes, John Butler, Rebecca S. Pepe, Hemant Phatak, and Karim El‐Kersh contributed to and approved the final version of the protocol. Lia N. Pizzicato and Samuel Governor conducted all statistical analyses. Lia N. Pizzicato, Vijay R. Nadipelli, Samuel Governor, Jianbin Mao, Stephan Lanes, John Butler, Rebecca S. Pepe, Hemant Phatak, and Karim El‐Kersh reviewed and interpreted results. Lia N. Pizzicato drafted the first draft of the manuscript. Vijay R. Nadipelli, Samuel Governor, Jianbin Mao, Stephan Lanes, John Butler, Rebecca S. Pepe, Hemant Phatak, and Karim El‐Kersh contributed to and approved the final version of the manuscript.

## CONFLICTS OF INTEREST

Authors Vijay R. Nadipelli, Jianbin Mao, John Butler, and Hemant Phatak are employees of Acceleron Pharma Inc., a subsidiary of Merck & Co., Inc., Rahway, NJ, USA, which funded this study. Karim El‐Kersh provided consultative services to Acceleron Pharma Inc., a subsidiary of Merck & Co., Inc., Rahway, NJ, USA, served on advisory boards for J&J Actelion, and United Therapeutics, received institutional research funding from J&J Actelion and United Therapeutics. Lia N. Pizzicato, Samuel Governor, Stephan Lanes, and Rebecca S. Pepe are employees of HealthCore Inc. (Wilmington, DE), which was contracted by Acceleron Pharma Inc., a subsidiary of Merck & Co., Inc., Rahway, NJ, USA, to perform this study.

## ETHICS STATEMENT

Researchers accessed data in the format of a limited data set for which data use agreements were in place with the covered entities in compliance with the Health Insurance Portability and Accountability Privacy Rule. Because this study was a secondary data analysis using a limited dataset, Intistutional Review Board approval was not required in accordance with HealthCore's Federal Wide Assurance.
